# A systematic review and meta-analysis of x-ray therapy versus proton beam therapy for pediatric central nervous system germ cell tumors: TRP-Germinoma 2025

**DOI:** 10.1007/s10147-025-02863-6

**Published:** 2025-08-21

**Authors:** Sho Hosaka, Masashi Mizumoto, Hiroko Fukushima, Takashi Iizumi, Takashi Saito, Masako Inaba, Ryoko Suzuki, Yinuo Li, Kei Nakai, Shosei Shimizu, Yoshiko Oshiro, Kazushi Maruo, Hidetoshi Takada, Hideyuki Sakurai

**Affiliations:** 1 Department of Pediatrics, University of Tsukuba Hospital, Tsukuba, Ibaraki 305-8576 Japan; 2 Department of Radiation Oncology, University of Tsukuba, Tsukuba, Ibaraki 305-8576 Japan; 3 Department of Child Health, Institute of Medicine, University of Tsukuba, Tsukuba, Ibaraki 305-8576 Japan; 4 Department of Pediatric Radiation Therapy Center/Pediatric Proton Beam Therapy Center, Hebei Yizhou Cancer Hospital, Zhuozhou City, 072750 China; 5 Department of Radiation Oncology, Tsukuba Medical Center Hospital, Tsukuba, Ibaraki 305-8558 Japan; 6 Department of Biostatistics, Institute of Medicine, University of Tsukuba, Tsukuba, Ibaraki 305-8576 Japan; 7 Proton Medical Research Center, University of Tsukuba, 1-1-1 Tennoudai, Tsukuba, Ibaraki 305-8575 Japan

**Keywords:** Germ cell tumor, Germinoma, Proton beam therapy, Radiotherapy, Meta-analysis

## Abstract

**Introduction:**

Central nervous system germ cell tumors (CNS-GCTs) are rare pediatric tumors, categorized into germinomas and non-germinomatous germ cell tumors (NGGCTs). Proton-beam therapy (PBT) has been introduced as an alternative to X-ray therapy (XRT) for minimizing radiation exposure to normal brain tissue, but evidence comparing these treatment modalities is limited.

**Methods:**

A systematic review and meta-analysis following PRISMA guidelines was conducted using PubMed for studies published between 1990 and 2022. Studies reporting overall survival (OS) and progression-free survival (PFS) for CNS-GCTs treated with PBT or XRT were included. Random-effects meta-analyses compared 3- and 5-year OS and PFS between treatment modalities.

**Results:**

Forty-one studies were selected, with 36 on XRT and five on PBT. In germinoma patients, no significant differences were found between XRT and PBT for 3-year OS (95.3% vs 97.8%, *p* = 0.3158), 5-year OS (94.8% vs 97.8%, *p* = 0.3088), 3-year PFS (90.7% vs 97.1%, *p* = 0.1045), or 5-year PFS (89.2% vs 91.7%, *p* = 0.4676). The collected data were insufficient to evaluate PBT in NGGCTs.

**Conclusion:**

PBT and XRT showed comparable survival outcomes in germinoma. Further research is required to explore PBT’s potential benefits in preserving cognitive function or reducing secondary cancers.

## Introduction

Central nervous system germ cell tumors (CNS-GCTs) account for less than 1% of all central nervous system tumors in children. CNS-GCTs can be classified into two groups: “pure” germinomas, and the more aggressive non-germinomatous germ cell tumors (NGGCTs). It is well known that radiotherapy plays an indispensable role in the treatment for CNS-GCTs, and multiple studies attempting to omit radiotherapy have reported failure in the treatment of germinoma [1, 2].

Recent advances in treatment modalities, such as proton-beam therapy (PBT), have broadened the choice for patients with various head and neck cancers [3, 4]. Reducing the radiation exposure to normal brain tissue in children can help preserve cognitive functions [5, 6], and thus the physical properties of PBT make it an attractive option for patients with CNS-GCT. However, due to the rarity of pediatric CNS-GCTs, evidence concerning the efficacy of PBT in comparison with conventional X-ray therapy (XRT) is scarce. Moreover, this type of tumor is relatively more common in Asia, particularly in Japan, where several institutions actively utilize proton beam therapy. Therefore, evaluating the outcomes of PBT in this regional context is highly relevant to both national and international oncology communities. To this end, we conducted a meta-analysis based on a comprehensive literature review.

## Methods

This meta-analysis was conducted following the principles and recommendations of the Preferred Reporting Items for Systematic Reviews and Meta-Analyses (PRISMA) statement [7]. PubMed was searched using the terms (germinoma OR germ cell) AND (radiotherapy OR proton) AND (children OR pediatrics) AND (brain), for articles published between 1990 and 2022. Only articles written in English were selected. Two reviewers independently screened all retrieved articles and selected papers using the following criteria: (1) Only articles related to pediatric CNS-GCT were selected from the search results. (2) Using the abstracts, studies with ten or more patients per treatment modality, and those that reported pathological diagnoses were then selected. (3) Lastly, based on a full text search, articles that reported overall survival (OS) and progression-free survival (PFS) for either modality were selected. In cases of multiple reports from the same research group covering overlapping periods, only the most recent studies were included. The following data were extracted from the selected articles: author, year, pathology (germinoma or NGGCT), number of cases, age, gender, metastatic status, treatment modality, treatment dose, status of craniospinal irradiation (CSI), chemotherapy status, follow-up period, OS, and PFS. Since whole ventricular or whole brain irradiation is considered the standard of care for germinoma, studies that used only local irradiation were omitted from the search.

Random effects meta-analyses of OS and PFS rates for each treatment modality were performed to generate forest plots. Data were extracted separately for germinoma and NGGCT. For studies with missing accuracy data, missing values were imputed using information on the number of cases, size of the risk set each year, and mean dropout rate. Heterogeneity in each meta-analysis was assessed by I-square statistics. Random-effects meta-regressions with modality as the explanatory variable were performed for each outcome to compare across modalities. All analyses were performed using R software (R Core Team, Vienna, Austria) and its meta packages [8].

## Results

**Fig. 1:**
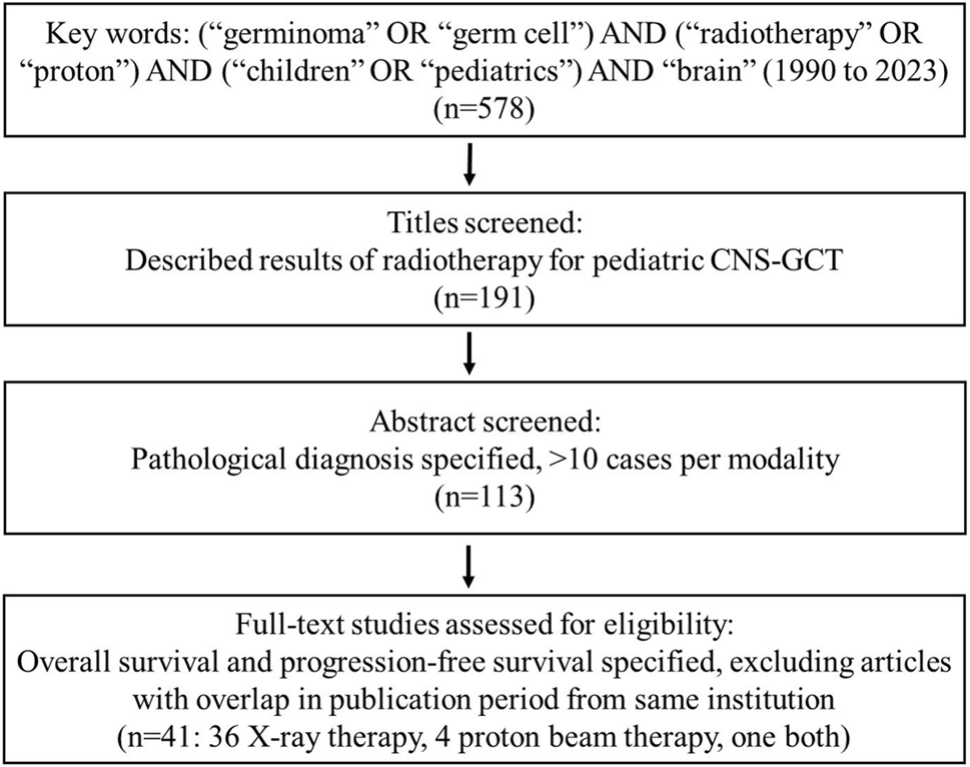
Flow diagram describing the selection of articles

The screening process and outcome of the selected articles are depicted in Fig. 1. Ultimately, 41 articles were eligible [9–49], of which 36 focused on XRT, 4 on PBT, and one on both. Four were prospective studies, and the rest were retrospective. Due to the limited number of articles describing treatment outcomes for PBT, data from an in-house registry describing 15 patients with germinoma were added to the analysis.

**Fig. 2:**
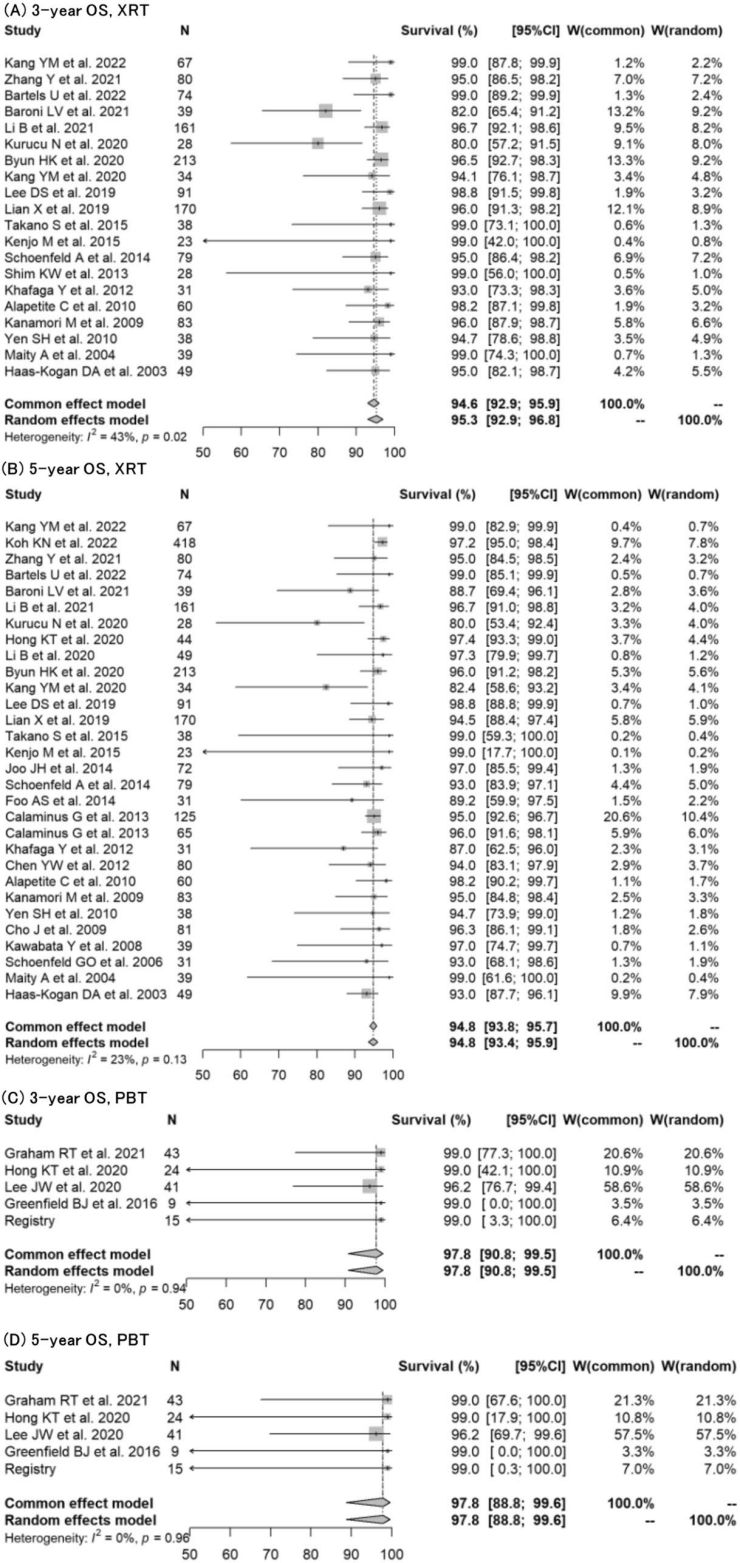
Forest plots describing overall survival for germinoma, comparing XRT with PBT. **A** 3-year OS for XRT [9, 12, 14, 15, 17, 18, 20–23, 26, 27, 30, 33, 34, 41, 43, 44, 47, 49]. **B** 5-year OS for XRT [9–12, 14, 15, 17–23, 25–30, 33, 34, 37, 38, 40–44, 47]. **C** 3-year OS for PBT [16, 24, 25, 39]. **D** 5-year OS for PBT [16, 24, 25, 39]

**Fig. 3:**
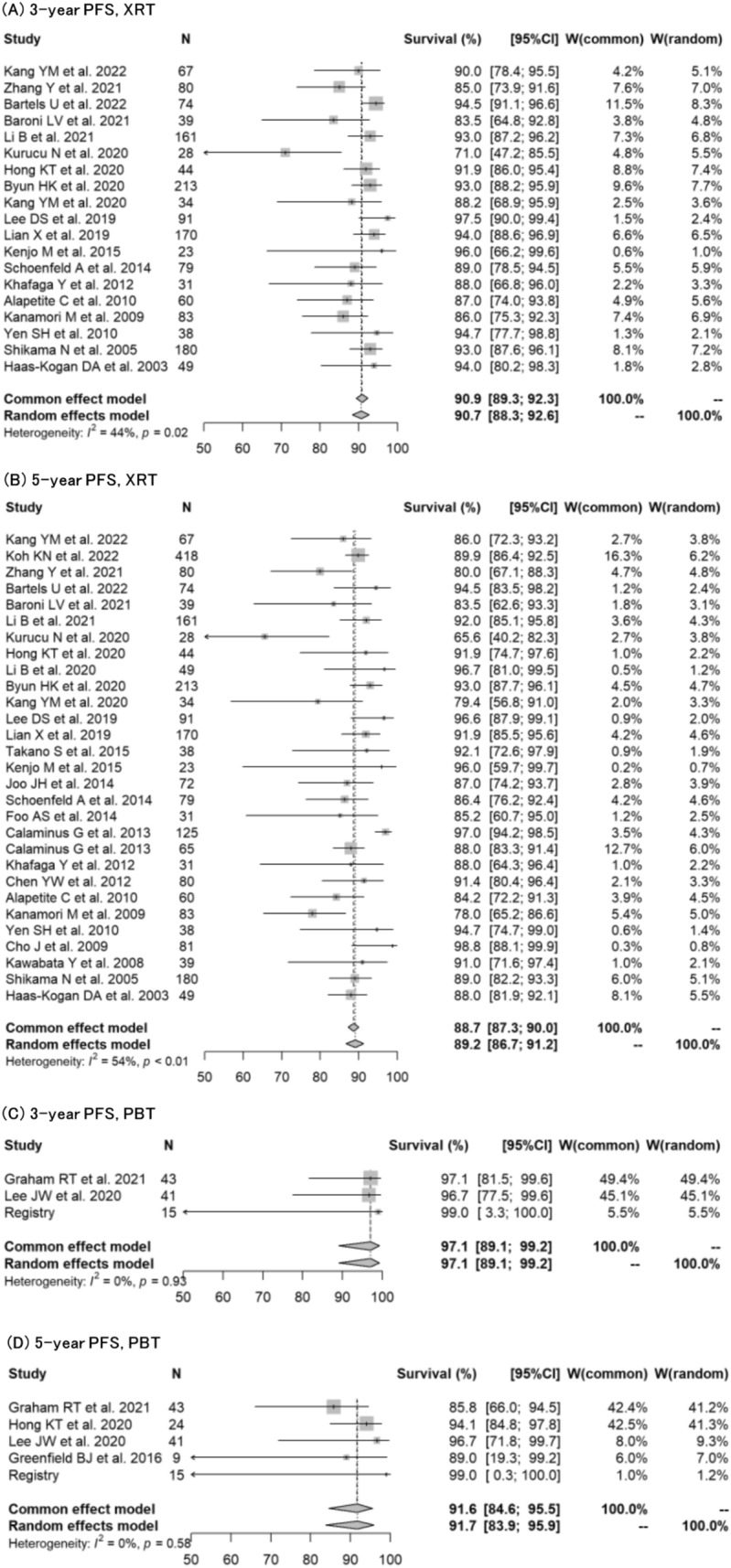
Forest plots describing progression-free survival for germinoma, comparing XRT with PBT. **A** 3-year PFS for XRT [9, 12, 14, 15, 17, 21–23, 25–27, 30, 33, 34, 41, 43, 44, 47, 48]. **B** 5-year PFS for XRT [9–12, 15, 17, 20–23, 26–28, 30, 33, 34, 37, 38, 40–44, 47, 48]. **C** 3-year PFS for PBT [16, 24]. **D** 5-year PFS for PBT [16, 24, 25, 39]

Regarding germinoma, forest plots for 3-year OS, 5-year OS, 3-year PFS, and 5-year PFS were created for each treatment modality (XRT or PBT) (Figs. 2, 3). The 3-year OS (XRT vs PBT) were 95.3% (95% CI: 92.9%−96.8%) vs 97.8% (95% CI: 90.8%−99.5%), *p* = 03158; and the 5-year OS (XRT vs PBT) were 94.8% (95% CI: 93.4%−95.9%) vs 97.8% (95% CI: 88.8%−99.6%), *p* = 0.3088, respectively. The 3-year PFS (XRT vs PBT) were 90.7% (95% CI: 88.3%−92.6%) vs 97.1% (95% CI: 89.1%−99.2%), *p* = 0.1045; and the 5-year PFS (XRT vs PBT) were 89.2% (95% CI: 86.7%−91.2%) vs 91.7% (95% CI: 83.9%−95.9%), *p* = 0.4676, respectively.

**Fig. 4:**
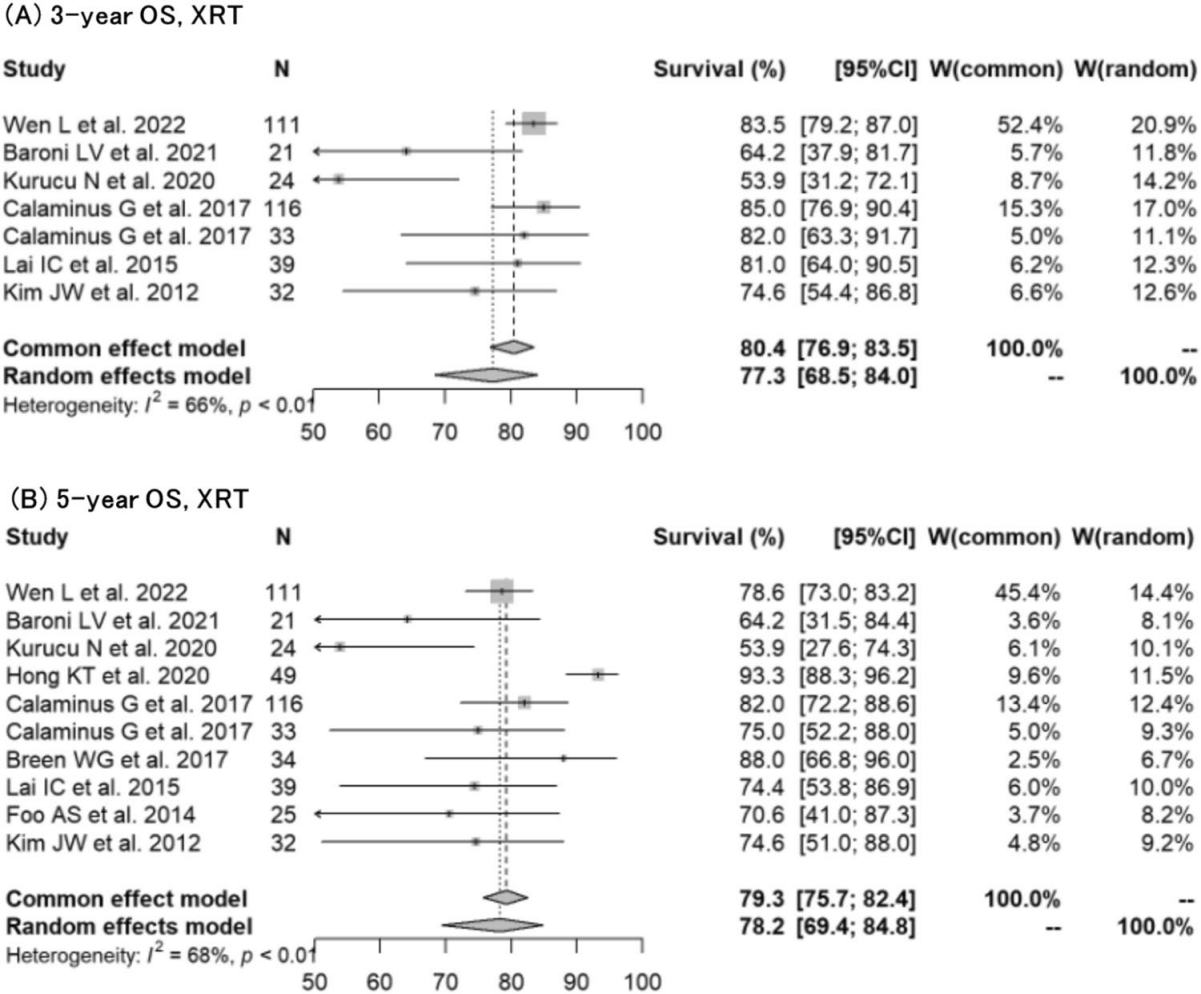
Forest plots describing overall survival for NGGCT treated with XRT. **A** 3-year OS [13, 14, 21, 22, 36, 45]. **B** 5-year OS [11, 13, 14, 22, 25, 32, 35, 36, 45]

**Fig. 5:**
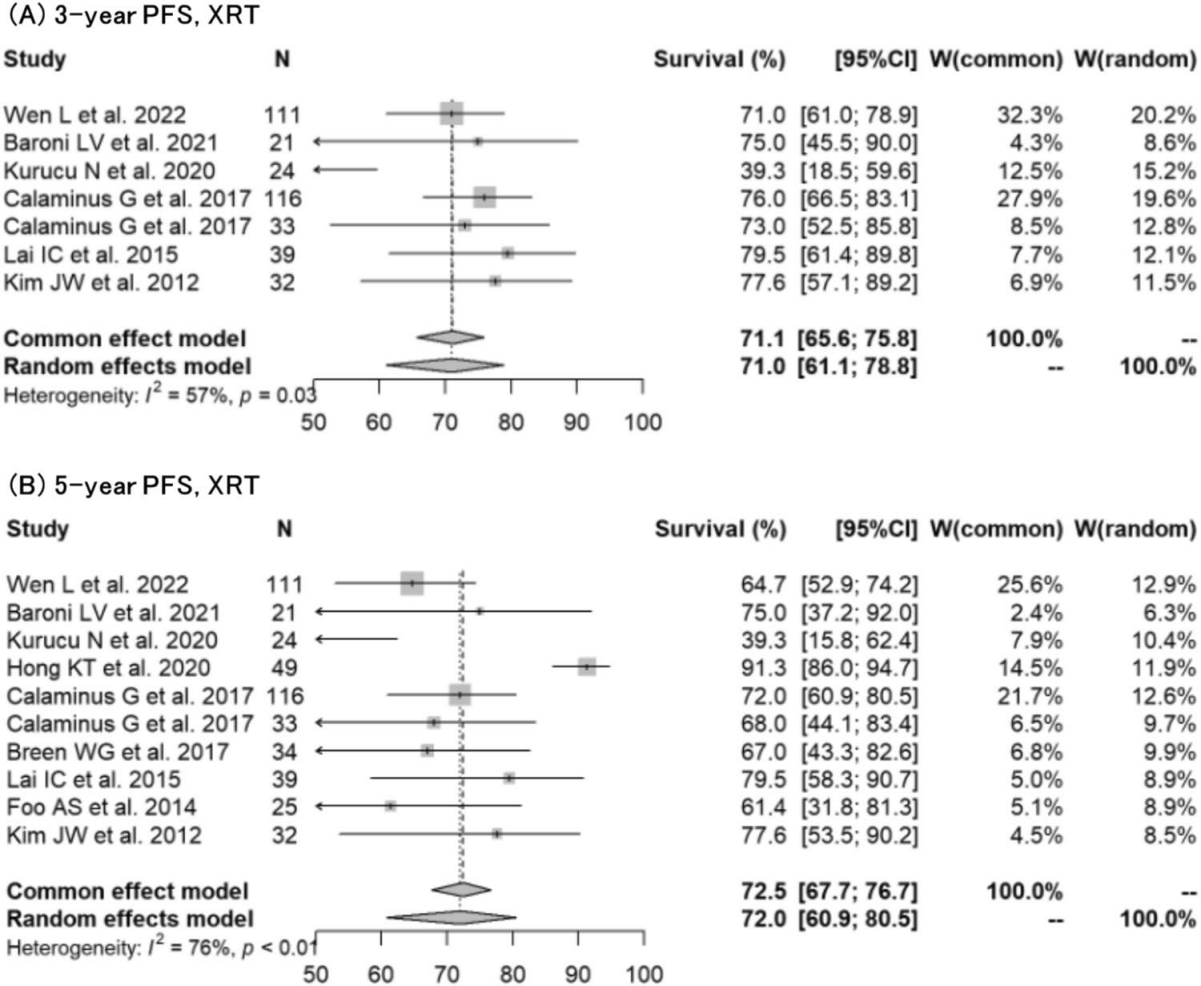
Forest plots describing progression-free survival for NGGCT treated with XRT. **A** 3-year PFS [13, 14, 21, 22, 36, 45]. **B** 5-year PFS [11, 13, 14, 22, 25, 32, 35, 36, 45]

Regarding NGGCT, forest plots for 3-year OS, 5-year OS, 3-year PFS, and 5-year PFS were created for XRT (Figs. 4, 5). The rates could not be calculated for PBT due to insufficient data. The 3-year OS and 5-year OS were 77.3% (95% CI: 68.5%–84.0%) and 78.2% (95% CI: 69.4%–84.8%), respectively. The 3-year PFS and 5-year PFS were 71.0% (95% CI: 61.1%–78.8%) and 72.0% (95% CI: 60.9%–80.5%), respectively.

Random-effects meta-regression analysis with modality as an explanatory variable was then adjusted with age, gender, and chemotherapy status. Treatment dose and the irradiation field (CSI status) were unaccounted for due to the lack of comparable data. Regarding germinoma, treatment modality, age, gender, and chemotherapy status did not significantly affect survival (Table 1). Regarding NGGCT, the addition of chemotherapy was associated with better 3-year OS (*p* = 0.0406) and 3-year PFS (*p* = 0.0078), but not with 5-year OS (*p* = 0.0732) or 5-year PFS (*p* = 0.0653). Treatment modality, age, and gender were not significantly associated with any of the survival outcomes (Table 2).

**Table 1:** Meta regression analysis of predictive factors for survival of germinoma

Factors	Coefficient	SE	Lower CL	Upper CL	Z Value	*p* Value
3y-OS						
Modality (PBT vs XRT)	−0.7285	0.9297	−2.5508	1.0938	−0.7835	0.4333
Age	−0.2402	0.1382	−0.5111	0.0308	−1.7372	0.0823
Gender (Male ratio)	−0.0310	0.0180	−0.0662	0.0043	−1.7234	0.0848
Chemotherapy	0.0061	0.0079	−0.0094	0.0217	0.7717	0.4403
5y-OS						
Modality (PBT vs XRT)	−0.4988	0.9961	−2.4511	1.4534	−0.5008	0.6165
Age	−0.1656	0.1073	−0.3759	0.0447	−1.5437	0.1227
Gender (Male ratio)	−0.0059	0.0169	−0.0390	0.0272	−0.3468	0.7287
Chemotherapy	−0.0041	0.0047	−0.0134	0.0052	−0.8678	0.3855
3y-PFS						
Modality (PBT vs XRT)	−1.0214	0.7603	−2.5114	0.4687	−1.3435	0.1791
Age	09.1655	0.0977	−0.3570	0.0259	−1.6950	0.0901
Gender (Male ratio)	0.0009	0.0147	−0.0280	0.0297	0.0581	0.9537
Chemotherapy	−0.0026	0.0050	−0.0125	0,0072	−0.5276	0.5978
5y-PFS						
Modality (PBT vs XRT)	−0.1797	0.5524	−1.2625	0.9031	−0.3253	0.7449
Age	−0.0578	0.0797	−0.2139	0.0983	−0.7259	0.4679
Gender (Male ratio)	0.0069	0.0142	−0.0210	0.0348	0.4836	0.6287
Chemotherapy	0.0020	0.0040	−0.0058	0.0098	0.5026	0.6153

**Table 2:** Meta regression analysis of predictive factors for survival of NGGCT

Factors	Coefficient	SE	Lower CL	Upper CL	Z Value	*p* Value
3y-OS						
Modality (PBT vs XRT)	−0.725	1.0137	−2.7119	1.2618	0.4745	0.4745
Age	−0.0825	0.117	−0.3118	0.1468	0.4806	0.4806
Gender (Male ratio)	−0.0115	0.0316	−0.0734	0.0504	0.7160	0.7160
Chemotherapy	−0.0475	0.0232	−0.0929	−0.0020	0.0406	0.0406
5y-OS						
Modality (PBT vs XRT)	−0.2039	0.8333	−1.8372	1.4294	−0.2447	0.8067
Age	−0.0710	0.1406	−0.3466	0.2046	−0.5047	0.6137
Gender (Male ratio)	−0.0004	0.0278	−0.0540	0.0548	0.0143	0.9886
Chemotherapy	−0.0574	0.0321	−0.1203	0.0054	−1.7915	0.0732
3y-PFS						
Modality (PBT vs XRT)	−0.8502	0.6625	−2.1487	0.4483	−1.2834	0.1994
Age	0.0567	0.0936	−0.1268	0.2402	0.6058	0.5446
Gender (Male ratio)	−0.0364	0.0256	−0.0865	0.0137	−1.4250	0.1542
Chemotherapy	−0.0493	0.0185	−0.0856	−0.0130	−2.6591	0.0078
5y-PFS						
Modality (PBT vs XRT)	−0.6803	0.8489	−2.3441	−.9834	−0.8014	0.4229
Age	0.0769	0.1427	−0.2028	0.3565	0.5387	0.5901
Gender (Male ratio)	−0.0298	0.0275	−0.0836	0.0241	−1.0834	0.2786
Chemotherapy	−0.0592	0.0321	−0.1222	0.0038	−1.8431	0.0653

## Discussion

CNS-GCTs, namely germinomas, are known for their potential to spread via the cerebrospinal fluid or directly into the brain parenchyma, and thus chemotherapy and radiotherapy are the mainstay of treatment [50]. Previous attempts to replace radiotherapy with chemotherapy led to higher relapse rates [1, 2], and radiotherapy is now considered to play a central role in the treatment of CNS-GCTs. Nevertheless, as the late adverse effects of radiotherapy on the childhood brain are well known [51], various efforts are made to reduce the radiation dosage, with PBT being one of them.

Since germinoma is known to invade deeply into the surrounding brain parenchyma, the limited irradiation field produced by PBT may raise concerns about local control and peri-field relapse. However, in the treatment of CNS-GCTs, local control is not often reported separately, and progression-free survival is generally used as a surrogate endpoint. The comparable PFS outcomes between PBT and XRT suggest that local disease control is not compromised with the use of proton therapy. This finding indirectly supports the appropriateness of current clinical target volume (CTV) definitions for PBT, despite its highly conformal nature.

A covariate adjusted random-effect regression analysis detected a slight survival advantage with the addition of chemotherapy for NGGCT (3-year OS, *p* = 0.0406; 3-year PFS, *p* = 0.0078). This is not surprising since NGGCTs are generally associated with a worse prognosis, and patients usually receive multimodal treatment including intense chemotherapy. No differences were detected for the other variables, including age, gender, or treatment modality. CNS-GCTs are relatively more common in East Asia, and therefore, evaluating the outcomes of PBT in this regional context is highly relevant to both national and international oncology communities.

This study has several limitations mostly due to the rarity of pediatric CNS-GCTs, especially NGGCTs. This makes it challenging to conduct prospective studies, and indeed, most of the selected articles were retrospective, which limits the quality of this study. Our results were not adjusted for the radiation portals or treatment dose, due to insufficient information about individual cases in the selected articles. Although whole ventricular irradiation with 18 to 23.4 Gy is considered to be the standard of care in germinoma [50], patients with basal ganglia tumors may receive whole brain irradiation while patients with spinal dissemination may receive CSI, making it difficult to control for the radiation portal. While we attempted to capture standard treatment approaches by excluding studies that used only focal irradiation, the included studies often employed a mix of radiation fields such as whole brain irradiation, whole ventricular irradiation, and craniospinal irradiation. These variations were not consistently reported nor stratified in the outcome data, precluding further subgroup analysis. This heterogeneity in treatment fields and prescribed doses likely confounds the comparison between XRT and PBT, and represents a major limitation of this study.

Other important issues surrounding the treatment of pediatric CNS-GCTs include the adverse effects on the brain, such as impaired cognitive functions or secondary cancers. Again, we were unable to directly compare treatment-associated toxicity or normal tissue exposure between PBT and XRT, since most of the selected articles in this study did not describe adverse effects or outcome data according to radiation field or dosage. Among the 41 studies included, 18 mentioned secondary brain tumors or cerebrovascular events. Of these 18 studies, 14 provided sufficient details about the radiation modality for 31 events. In all such cases, the patients had received either whole-brain (WB) or craniospinal irradiation (CSI); no secondary tumors or cerebrovascular events were reported after whole ventricular (WV) irradiation. Moreover, among these 14 studies, 12 used XRT (*n* = 945), resulting in 23 events; one used PBT (*n* = 20), resulting in one event; and one used both modalities (*n* = 127), resulting in 7 events, although the events in this study could not be attributed to either modality. Consequently, the number of such adverse events was low, and the heterogeneity in radiation modality, field size, dose, and follow-up period among the studies precluded any meaningful comparison between X-ray therapy and proton beam therapy in terms of secondary malignancy risk. This represents a major limitation and underscores the need for standardized toxicity reporting in future research, which should aim to separately evaluate modality-specific adverse events, especially long-term effects such as neurocognitive outcomes and secondary malignancies which are of particular concern in pediatric patients receiving cranial irradiation. Nonetheless, although we could not assess late adverse events such as secondary malignancies in this analysis, proton therapy has been associated with a lower relative risk of secondary cancers in other pediatric populations [52]. This potential benefit, along with the advantage of preserving cognitive function, warrants further investigation in future prospective studies.

## Conclusion

A meta-analysis of radiotherapy for pediatric CNS-GCT revealed that PBT did not differ from XRT in terms of overall survival and progression-free survival. A direct comparison of toxicity or normal tissue exposure between PBT and XRT could not be performed due to insufficient data. Future prospective studies will be needed to clarify the toxicity profiles and protective effects of PBT on brain function.

## Data Availability

All data generated or analyzed during this study are included in this article. Further enquiries can be directed to the corresponding author.

## Abbreviations

CNS: Central nervous system
CSI: Cranio-spinal irradiation
GCT: Germ cell tumor
NGGCT: Non-germinomatous germ cell tumor
OS: Overall survival
PBT: Proton beam therapy
FS: Progression-free survival
RT: X-ray therapy
